# Associations of an empirical dietary pattern with cardiometabolic risk factors in Malaysian adolescents

**DOI:** 10.1186/s12986-020-00447-x

**Published:** 2020-04-07

**Authors:** Nor Aishah Emi, Wan Ying Gan, Zalilah Mohd Shariff, Azriyanti Anuar Zaini, Nurainul Hana Shamsuddin, Mahenderan Appukutty, Geeta Appannah

**Affiliations:** 1 Department of Nutrition and Dietetics, Faculty of Medicine and Health Sciences, Universiti Putra Malaysia, Serdang, Selangor Malaysia; 2 Department of Paediatrics, Faculty of Medicine, University of Malaya, Kuala Lumpur, Malaysia; 3 Department of Family Medicine, Faculty of Medicine and Health Sciences, Universiti Putra Malaysia, Serdang, Selangor Malaysia; 4 Sports Science Programme, Faculty of Sports Science and Recreation, Universiti Teknologi MARA, Shah Alam, Selangor Malaysia

**Keywords:** Dietary patterns, Dyslipidemia, Cardiometabolic risk factors, Childhood obesity, Malaysian adolescent

## Abstract

**Background:**

This study aimed to identify a dietary pattern (DP) characterised mainly by high intakes of free sugar and other nutrients hypothesised to be associated with obesity such as dietary energy density (DED), percentage of energy from total fat and fibre density in adolescents from three southern states of Peninsular Malaysia, and its associations with cardiometabolic risk factors.

**Methods:**

This is a cross-sectional study among 335 adolescents who provided both dietary information assessed using a validated food frequency questionnaire (FFQ) and biochemical parameters including lipid profile, blood glucose, serum insulin and homeostatic model assessment-insulin resistance (HOMA-IR). Anthropometric measurements included weight (kg), height (cm) and waist circumference (cm), while body mass index (BMI) in kg/m^2^ was estimated, respectively. Reduced rank regression (RRR) identified a DP with percentage of energy from sugar and total fat, DED and fibre density intake as response variables.

**Results:**

The identified ‘high sugar, high fibre, high DED and low fat’ DP was characterised by high intakes of sugar-sweetened beverages, fruits, sweets and low intakes of meat and cereal. Adolescents in the highest tertile of the identified DP had about 3.0 (OR = 2.7; 95%CI: 1.3, 5.6) and 2.0 (OR = 1.9; 95%CI: 1.0, 3.5) times higher odds of having dyslipideamia or elevated total cholesterol and LDL-cholesterol level, respectively compared to adolescents in the lowest tertile DP after adjusting for sex, school location, maternal education, physical activity, dietary misreporting and BMI z-score. This DP was not significantly associated with overweight and obesity.

**Conclusions:**

Higher adherence to a DP characterised mainly by free sugars and DED was associated with greater odds of having dyslipideamia, elevated total cholesterol and LDL-cholesterol levels in Malaysian adolescents.

## Background

There is strong evidence that the precursor of adult cardiovascular diseases (CVD) begins in childhood, with obesity being the main risk factor among other cardiometabolic risk factors such as insulin resistance and dyslipideamia [1–4]. In line with that, a rapid escalating trend of overweight and obesity among Malaysian adolescents was reported in a recent National Health and Morbidity Survey as well as in a systematic review by Majid et al. in 2015 [5–7].

Over the past decades, accumulated number of evidences have been supporting the role of diet as one of the most important modifiable factor for the prevention of CVD including during childhood and adolescence stages [8]. Adolescence is a period of marked physiological, biological and psychological changes, and may be a key time period for the establishment of lifelong dietary habits [9]. Therefore, early identification of specific dietary habits during adolescence might be beneficial for the prevention of CVD later in life. However, there is very limited information on dietary habits and their relationships with cardiometabolic risk factors among Malaysian young people.

Having said that, a few observational studies in the Western countries have evaluated associations between empirically derived dietary patterns (DPs) and cardiometabolic risk factors in adolescents [10–13]. For example, two pregnancy cohort studies in Australia and United Kingdom (UK) reported that a DP characterised by food intakes high in dietary energy density (DED), high fat and low fibre was associated greater adiposity and cardiometabolic risk factors during adolescence in 2010 and 2015, respectively [11, 12]. Because of emerging importance of dietary sugar in the development of obesity, an extension to the UK study was performed in 2016 [12, 14]. It was reported that adolescents with greater adherence to a DP characterised by high intakes of dietary fats and sugar were found to be associated with greater adiposity. Dietary fats and sugar might be associated with overconsumption of energy, and therefore may act as dietary risk factors for the development of obesity and CVD [14, 15].

However, given the differences in cultures and dietary habits across the world, it is highly possible that much are remain to be known on dietary patterns and health outcomes, particularly of those countries in the East. To date, no studies have assessed empirical DPs in relation to cardiometabolic risk factors among Malaysian adolescents. In this study, we hypothesised that a DP characterised by response variables linked to obesity namely free sugar, dietary fat, DED, and fibre would be associated with cardiometabolic risk factors among adolescents in Malaysia.

## Methods

### Study design and sample

This cross-sectional study was conducted from August to November 2016 in several public secondary schools that were randomly selected within three states in the southern region of Peninsular Malaysia namely Negeri Sembilan, Melaka and Johor. A probability proportionate sampling design was used for selecting these schools [16]. This was done by obtaining a complete list of public secondary schools and the estimated number of adolescents aged 13 years old in each school (sampling frame) from the Ministry of Education (MOE) Malaysia. In Malaysia, adolescents usually enter public secondary schools at the age of 13 years. This age is important as it represents a key transition period from primary to secondary education levels that correlates with major pubertal changes and increases in personal autonomy (e.g. less parental control and more social freedom), both of which may affect food choices [17]. A total of 24 selected schools were approached and out of these, only 21 schools agreed to participate.

Sample size for this study was estimated using a formula for single cross-sectional survey. The calculation for minimum sample size was based on the prevalence of obesity in children (20.9%) [18]. After taking into account a 20% response rate and a design effect of 2, a total number of 1000 adolescents was estimated for this study. Out of the estimated 1000 adolescents, a total of 933 agreed to participate (93% of response rate). Information on anthropometric, physical activity, dietary assessment and biochemical measurement was provided by 930, 793, 585 and 507 adolescents, respectively. Out of these, 336 adolescents provided both cardiometabolic and valid dietary data while 582 adolescents provided both the anthropometric and dietary data. Supplementary Figure 1 illustrates the number of adolescents who provided anthropometric, biochemical, dietary and physical activity data in this study.

Full ethical approval was obtained from the Ethics Committee for Research Involving Human Subjects (JKEUPM) of Universiti Putra Malaysia (UPM) (Reference number: FPSK (EXP16) P031). Approval to conduct this study in the selected schools were obtained from the Ministry of Education Malaysia, state education departments and selected schools. The study respondents and their parents provided written consent before the commencement of the study.

### Patient and public involvement

Prior to the recruitment, adolescents in the selected schools were screened for their eligibility. Adolescents aged 13 years old during the data collection comprised of both sexes, with adequate ability to read and understand Malay or English language were eligible to participate in the study. However, adolescents with physical disabilities and chronic conditions were not eligible to participate in this study. Eligible adolescents were invited to take part in this study and were given a copy of study information sheet. Interested adolescents were asked to sign an assent form after being clearly briefed on all the procedures required for the study. An informed consent form was sent to their parents for further approval. Parents who agreed with the study involvement were asked to complete the parental questionnaire at home and return it to the study researcher through their children. During the second visit to the school, a research team comprised of a dietitian, a medical doctor, and two research enumerators collected various data i.e. socio-demography, dietary, physical activity, anthropometric and biochemical measurements from the study adolescents.

### Dietary assessment

A validated adolescent food frequency questionnaire (FFQ) was used to assess dietary intakes in the past 12 months and details of this FFQ has been previously described [19]. In brief, the MyUM FFQ was originally developed by a group of researchers from a contemporaneous study to that of this study in Universiti Malaya (UM) [17]. It comprised of 195 food items and is a self-administered questionnaire designed especially for Malaysian secondary school adolescents aged between 13 to 18 years. Before administering the FFQ, step-by-step instructions on how to fill in the questionnaires were given to all participants by the study researchers. As an aid to estimate food intake, the participants were provided with a flipchart on household measurements. The food intake frequency and portion size of each food item were recorded by the adolescents. The average frequency for consumption of each food item over the past year was recorded as ‘never’, ‘1-3 times per month’, ‘one time a week’, ‘2-4 times per week’, ‘5-6 times per week’, ‘one time a day’, ‘2-3 times per day’, ‘4-5 times per day’, or ‘≥6 times per day’. The study researchers checked all the questionnaires upon submission to ensure that all fields were filled in.

The process of converting the FFQ raw data to daily energy and other nutrients were conducted manually using a standard conversion factor to estimate daily food intake based on the frequency of food consumption [20]. The estimation of daily food intake was analysed using the Nutritionist Pro software version 3.1 (Axxya Systems, USA). The dietary data of the food items which were included in the FFQ were derived from the Malaysian Food Composition [21, 22]. Adolescents whose overall dietary energy intake were outside the range of 400–8000 kcal (or 1674–33,472 kJ) were excluded from the DP analysis [23].

Dietary misreporting was estimated using the Goldberg eq. (2000) according to the ratio of energy intake (EI) to basal metabolic rate (BMR) [24]. BMR was calculated using a sex-specific formula for Malaysian adolescents aged 13 years [25]. Physical activity level (PAL) was set at 1.55 as majority of adolescents had been reported to have low physical activity levels [26]. The cut-off values for dietary misreporting was calculated based on the confidence limit of agreement between the ratio of EI to BMR and PAL. Adolescents with the ratio of EI to BMR ranging from 1.09 to 2.21 were considered as plausible reporters, otherwise, they were considered as under-reporters or over-reporters, accordingly. Variable on dietary misreporting was included as a potential covariate in statistical models.

### Dietary patterns

Reduced rank regression (RRR) analysis was used to derive empirical DPs using SAS software version 9.4 (SAS Institute, Cary, NC). The RRR is a statistical method to determine linear function of predictor variables (food groups) by maximising the explained variation in nutrients (response variables) related to the disease of interest [27]. All the food items from the FFQ were categorised into 13 food groups (g/d) based on their nutritional characteristics and were used as predictor variables “Supplementary Table 1” [13]. DED, percentage of energy from total fat intake, percentage of energy from total sugar intake and fibre density were selected as response variables. These selected response variables showed significant associations with obesity and other cardiometabolic risk factors in previous prospective studies in Australia and UK [11, 12].

DED was calculated by dividing total food energy (kJ) with total food weight (g) by excluding beverages [13]. Meanwhile, fibre density was determined by absolute fibre intake (g/d) divided by total daily energy intake (MJ) [13]. Percentages of energy from total fat and sugar intakes were expressed by dividing total energy intake from fat (kJ) or total energy intake from free sugar (kJ) by total energy intake (kJ), followed by a multiplication of 100 [13, 14]. In this study, dietary sugar was defined as short-chained carbohydrates known as monosaccharide and disaccharides presented naturally in foods such as fruits or in manufactured products such as refined sugar [28].

A separate RRR analysis was applied specifically to investigate any gender variations between the DPs. However, as the DPs derived for both males and females were similar in their factor loading of food groups, thus DPs derived for the total respondents were taken for further analysis. Each adolescent obtained an individual z-score for each DP derived; a higher z-score corresponded to a higher adherence to the identified DP. Z-scores for the identified DP were analysed continuously and categorically (tertiles) with the lowest tertile set as the reference category.

### Measurement of cardiometabolic risk factors

A digital scale (Tanita HD319, Japan) was used to measure the adolescents’ body weight in kilogram, while their body height in centimetre was measured using a stadiometer (Seca 206, Germany). A measuring tape (Seca 201, Germany) was used to measure the adolescents’ waist circumference (WC) at the midpoint between their lower border of the ribs and their upper border of the pelvis. All measurements were repeated twice to obtain a mean value for each variable which were then used in the analysis.

Adolescents’ BMI was estimated by using a standard formula, body weight (kg) divided by the squared measured body height (m^2^). In addition, BMI z-score for age and gender was calculated using the WHO Anthro Software [29]. Overweight and obesity were defined using the BMI z-scores, whereby values of more than one and two standard deviations indicated possible risk of overweight and obesity, respectively. Computed WC z-scores were utilised for the analyses of abdominal obesity and was defined according to the Malaysian WC centile [30].

Adolescents who consented for blood withdrawal were asked to fast overnight before venepuncture by a phlebotomist. A total of 10 ml of fasting blood was withdrawn from each adolescent for insulin, fasting blood glucose (FBG), total cholesterol (TC), triglycerides (TG), HDL-C and low-density lipoprotein cholesterol (LDL-C) analyses. Insulin resistance was estimated using Homeostasis Model Assessment (HOMA) [31]. FBG concentration was measured by hexokinase assay using reagent by ADVIA Chemistry Glucose Hexokinase_3 and insulin concentration was measured using two-site sandwich immunoassay by ADVIA Centaur Insulin assay (Siemens Healthcare Diagnostics Inc., Tarrytown NY, USA). TC and HDL-C concentration were measured by enzymatic endpoint method and elimination/catalase method, respectively using reagent by ADVIA Chemistry (Siemens Healthcare Diagnostics Inc., Tarrytown NY, USA). TG concentration was measured by enzymatic reaction method with Trinder endpoint using ADVIA TRIG_2 reagent (Siemens Healthcare Diagnostics Inc., Tarrytown NY, USA). LDL-C and HOMA was calculated using the Friedewald formula (1972) and standard formula by Metthews et al. (1985), respectively [32, 33].

Dyslipidaemia during adolescence was defined when either the study adolescents’ TC level was greater than or equal to 5.2 mmol/L or their LDL-C level was greater than or equal to 3.4 mmol/L [34]. Adolescents with abnormal biochemical values were classified if their biochemical parameter values were ≥ 5.60 mmol/L for blood glucose, ≥5.20 mmol/L for total cholesterol, ≤1.03 mmol/L for HDL-cholesterol, ≥4.12 mmol/L for LDL-cholesterol, ≥1.70 mmol/L for triglycerides, ≥25.0 uIU/mL for serum insulin and ≥ 4.0 unit for HOMA-IR level [34–37].

### Covariates

A set of parents’ and study adolescents’ socio-demographic information was collected in this study. Parents were requested to complete a self-administered parental questionnaire comprised of information on educational level, occupation and monthly income. Meanwhile, adolescent questionnaire included questions such as date of birth, ethnicity, religion and gender. Self-reported physical activity in the past seven days was assessed using Physical Activity Questionnaire for Older Children (PAQ-C) [38]. PAQ-C has been validated and showed acceptable validity and good internal consistency among Malaysian adolescents [39, 40].

### Statistical analysis

Descriptive data were presented in mean ± standard deviation (SD) for continuous data and in frequency (n) and percentage (%) for categorical data. Comparisons between genders were performed using independent t-test and chi-square test. Binary logistic regression analysis was conducted to evaluate association between tertiles (2nd tertile vs. 1st tertile and 3rd tertile vs. 1st tertile) of the DP z-scores and overweight or obesity, abdominal obesity, dyslipidaemia, elevated values of FBG, TC, LDL-C, TG, insulin, HOMA-IR and low HDL-C. Regression models were conducted for all the cardiometabolic parameters in male and female, separetely due to sex dimorphism and puberty-related differences in growth [41]. All the models were adjusted for covariates including gender, school location (rural and urban), mother’s educational level (no formal education or primary level, secondary school level and tertiary level), dietary misreporting, physical activity and BMI z-score (for biochemical parameters only). All the above-mentioned analyses were ran using IBM SPSS Statistics software version 23 (IBM Corporation, New York, US) and a *p*-value of < 0.05 was considered as statistically significant.

## Results

In this study, a total of 68% of adolescents were females and the predominant ethnic group was Malay (87%), while the rest were either Chinese (6.8%), Indian (4.7%) or belonged to other ethnicity groups (1.3%) such as *Iban* and *Kadazan*. Adolescents from urban and rural schools were 434 (46.5%) and 499 (53.5%), respectively. Only 6% of the adolescents were from schools of smaller size while the rest of the adolescents were from either medium or larger sized schools. A majority of adolescents’ parent reported to have a monthly income of less than RM 5, 228 (88.6%). Most number of the adolescents’ mothers completed their education at secondary school level (73.8%). Males scored significantly higher physical activity levels compared to females “Table 1”.

**Table 1:** Characteristics of study adolescents and their parents recruited from three southern states of Peninsular Malaysia

Characteristics	*n* (%)			p-value
	Male (***n*** = 300)	Female (***n*** = 633)	Total	
School location				
Urban	147 (49.0)	287 (45.3)	434 (46.5)	0.29
Rural	153 (51.0)	346 (54.7)	499 (53.5)	
School size				
Small (≤100 students)	19 (6.3)	39 (6.2)	58 (6.2)	0.93
Medium (101–199 students)	109 (36.3)	238 (37.6)	347 (37.2)	
Large (≥200 students)	172 (57.3)	356 (56.2)	528 (56.6)	
Ethnicity				
Malay	262 (87.3)	552 (87.2)	814 (87.2)	0.95
Chinese	21 (7.0)	42 (6.6)	63 (6.8)	
Indian	14 (4.7)	30 (4.7)	44 (4.7)	
Others	3 (1.0)	9 (1.4)	12 (1.3)	
Parental Income, *n* = 599^a^				
Below median	159 (88.3)	372 (88.8)	531 (88.6)	0.89
Above median	21 (11.7)	47 (11.2)	68 (11.4)	
Educational level of mother, *n* = 818				
No formal education/Primary	23 (9.5)	68 (11.8)	91 (11.1)	0.58
Secondary school	179 (74.3)	425 (73.7)	604 (73.8)	
Higher institution	39 (16.2)	84 (14.6)	123 (15.0)	
Physical activity total score, *n* = 793	2.77 (±0.72)	2.40 (±0.57)	2.52 (±0.64)	< 0.001
‘High sugar, high fibre, high DED and low fat’ DP z-score, *n* = 583	−0.15 (±1.14)	0.06 (±1.40)	0.00 (±1.33)	0.08

^a^Median monthly household income (RM 5, 228) based on Household Income and Basic Amenities Survey 2016. *p* < 0.05 considered significant. *DED* Dietary Energy Density, *DP* Dietary Pattern

The total prevalence of overweight or obesity and abdominal obesity in this study was 32.4 and 11.6%, respectively. There were no significant differences observed in BMI (kg/m^2^) and waist circumference (cm) between the genders. Males showed lower mean values for TC (mmol/L), HDL-C (mmol/L) and LDL-C (mmol/L) levels and a higher FBG (mmol/L) value compared to females (*P* < 0.05) “Table 2”.

**Table 2:** Anthropometric and biochemical characteristics of adolescents aged 13 years recruited from three southern states of Peninsular Malaysia

Characteristics	Male	Female	Total Mean (SD)	*p*-value
	Mean (SD)	Mean (SD)		
Height (cm), *n* = 930	155.4 (±8.5)	153.0 (±5.9)	153.8 (±6.9)	< 0.001
Weight (kg), *n* = 930	49.8 (±14.6)	48.6 (±13.1)	49.0 (±13.6)	0.20
BMI (kg/m^2^), *n* = 930	20.5 (±5.3)	20.6 (±5.0)	20.6 (±5.1)	0.65
BMI z-score, *n* = 930	0.37 (±1.57)	0.26 (±1.48)	0.29 (±1.51)	0.30
WC (cm), *n* = 929	66.7 (±13.5)	65.0 (±10.8)	65.5 (±11.7)	0.05
WC z-score, *n* = 929	0.10 (±1.15)	−0.05 (±0.92)	0.00 (±1.00)	0.03
Fasting Blood Glucose (mmol/L), *n* = 507	4.93 (±0.39)	4.83 (±0.42)	4.86 (±0.42)	0.02
Total cholesterol (mmol/L), *n* = 507	4.36 (±0.72)	4.71 (±0.79)	4.60 (±0.79)	< 0.001
HDL cholesterol (mmol/L), *n* = 507	1.48 (±0.32)	1.58 (±0.32)	1.55 (±0.33)	0.003
LDL cholesterol (mmol/L), *n* = 507	2.47 (±0.63)	2.73 (±0.70)	2.65 (±0.69)	< 0.001
Triglycerides (mmol/L), *n* = 507	0.88 (±0.54)	0.89 (±0.38)	0.89 (±0.44)	0.78
Serum insulin (uIU/mL), *n* = 507	14.14 (±9.07)	13.96 (±8.08)	14.02 (±8.38)	0.83
HOMA-IR (unit), *n* = 507	3.14 (±2.20)	3.03 (±1.83)	3.06 (±1.95)	0.55
	*n* (%)	*n* (%)	*n* (%)	*p*-value
Overweight/obesity, *n* = 930	103 (34.6)	198 (31.4)	301 (32.4)	0.11
Abdominal obesity, *n* = 929	35 (11.8)	73 (11.6)	108 (11.6)	0.92
Dyslipidaemia, *n* = 507	22 (14.3)	99 (28.0)	121 (23.9)	0.001^a^
Plausible dietary reporter, *n* = 585	54 (31.4)	117 (28.5)	171 (29.2)	0.11

^a^Fisher’s exact test. Overweight and obesity were defined using BMI z-scores of more than one and two standard deviations above the WHO growth standard median, respectively. WC z-score was computed and abdominal obesity was defined according to the Malaysian WC centile curves of equal or more than the 90th centiles [28]. Dyslipidaemia during childhood and adolescence was determined when the level of total cholesterol was greater than or equal to 5.2 mmol/L or their LDL-C level was greater than or equal to 3.4 mmol/L [30]. Plausible dietary reporter was adolescents with the ratio of energy intake to basal metabolic rate between 1.09 to 2.21 estimated using the Goldberg equation [22]. *BMI* Body Mass Index, *WC* Waist Circumference, *HDL-C* High Density Lipoprotein Cholesterol, *LDL-C* Low Density Lipoprotein Cholesterol, *HOMA-IR* Homeostatic Model Assessment of Insulin Resistance

### Dietary patterns

A total of four DPs was identified and the percentage of variations explained by all response variables were 35, 14, 9 and 1% for DP1, DP2, DP3 and DP4, respectively “Supplementary Table 2”. The first DP was chosen for further analysis as it explained the greatest proportion of the variation in all response variables (35%) and reflected a dietary pattern that may positively associated with greater obesity risk. The selected DP was positively correlated with DED (r = 0.39), percentage of energy from sugar (r = 0.64), fibre density (r = 0.61) and negatively correlated with percentage of energy from fat (r = − 0.26). The identified ‘high sugar, high fibre, high energy density and low fat’ DP was characterised by high intakes of sugar-sweetened beverages (SSB) (r = 0.83), fruits (r = 0.33), sweets (r = 0.26), low intakes of cereal and cereal-based dishes (r = − 0.22), as well as, meat and meat dishes (r = − 0.21).

### Dietary pattern and cardiometabolic risk factors

No significant associations were observed between the tertiles of DP z-scores and overweight or obesity as well as abdominal obesity among the study adolescents in both unadjusted and adjusted models “Tables 3 and 4”. Adolescents in the third tertile of the ‘high sugar, high fibre, high energy density and low fat’ DP z-scores had 2 times higher odds of having elevated TC level (OR = 2.3; 95%CI: 1.2, 4.3) and elevated LDL-C level (OR = 1.8; 95%CI: 1.0, 3.0), compared to adolescents in the first tertile of DP z-score “Table 3”. These associations were further increased after adjusting for confounding factors, whereby adolescents in the highest tertile of DP z-scores had about 3 (OR = 2.7; 95%CI: 1.3, 5.6) and 2 (OR = 1.9; 95%CI: 1.0, 3.5) times higher odds of having elevated TC and LDL-C levels, compared to adolescents in the lowest tertile of DP z-scores “Table 4”. Similarly, adolescents in the highest tertile of the identified DP-zscores showed 3 times (OR = 2.7; 95%CI: 1.3, 5.6) higher odds of having dyslipideamia compared to adolescents in the lowest tertile, after adjusting for confounders “Table 4”.

**Table 3:** Unadjusted associations [odds ratio (95% CI)] between ‘high sugar, high fibre, high energy density and low fat’ DP z-scores and cardiometabolic risk factors in adolescents aged 13 years recruited from three southern states of Peninsular Malaysia

Cardiometabolic risk factors	Male		Female		Total	
	2nd tertile vs 1st tertile	3rd tertile vs 1st tertile	2nd tertile vs 1st tertile	3rd tertile vs 1st tertile	2nd tertile vs 1st tertile	3rd tertile vs 1st tertile
Overweight/Obese, *n* = 582	0.9 (0.4,1.9)	0.7 (0.3,1.6)	0.9 (0.5,1.5)	0.8 (0.5,1.4)	0.9 (0.6,1.4)	0.8 (0.5,1.2)
Abdominal Obesity, *n* = 581	0.8 (0.3,2.7)	1.5 (0.5,4.5)	0.9 (0.5,1.9)	0.4 (0.19,1.0)	0.9(0.5,1.7)	0.7(0.4,1.3)
Dyslipidaemia, *n* = 336	0.7 (0.1,3.9)	2.9 (0.8,10.9)	1.9 (0.9,3.9)	2.0 (0.9,4.2)	1.7 (0.9,3.3)	2.3* (1.2,4.3)
Elevated blood glucose (≥5.60 mmol/L), *n* = 336	4.5 (0.4,45.7)	8.1 (0.9,73.8)	0.0 (0.0,0.0)	0.9 (0.2,3.8)	0.6 (0.1,2.5)	2.0 (0.6,6.1)
Elevated total cholesterol (≥5.20 mmol/L), *n* = 336	0.7 (0.1,3.9)	2.9 (0.8,10.9)	1.9 (0.9,3.9)	2.0 (0.9,4.2)	1.7 (0.9,3.3)	2.3* (1.2,4.3)
Lower HDL-cholesterol level (≤1.03 mmol/L), *n* = 336	0.7 (0.1,7.9)	3.0 (0.5,17.9)	0.3 (0.1,1.8)	0.5 (0.1,2.3)	0.4 (0.1,1.7)	1.0 (0.4,3.1)
Elevated LDL-cholesterol (≥4.12 mmol/L), *n* = 336	1.2 (0.4,3.1)	2.1 (0.8,5.4)	1.6 (0.9,3.1)	1.6 (0.8,3.0)	1.6 (0.9,2.6)	1.8* (1.0,3.0)
Elevated triglycerides (≥1.70 mmol/L), *n* = 336	0.7 (0.1,7.9)	0.7 (0.1,7.9)	2.7 (0.3,26.6)	4.9 (0.6,42.7)	1.4 (0.3,6.2)	2.2 (0.5,8.8)
Elevated serum insulin (≥25.0 uIU/mL), *n* = 336	1.4 (0.2,10.6)	4.0 (0.7,22.1)	0.6 (0.2,2.0)	0.6 (0.2,2.1)	0.8(0.8,2.1)	1.2 (0.5,3.0)
Abnormal HOMA-IR level (≥4.0 unit), *n* = 336	2.4 (0.7,22.1)	4.1* (1.1,14.8)	1.2 (0.6,2.5)	0.9 (0.4,1.9)	1.5 (0.8,2.8)	1.4 (0.7,2.6)

*p < 0.05 is considered significant. *HDL-C* High Density Lipoprotein Cholesterol, *LDL-C* Low Density Lipoprotein Cholesterol, *HOMA-IR* Homeostatic Model Assessment of Insulin Resistance

**Table 4:** Adjusted associations [odds ratio (95% CI)] between ‘high sugar, high fibre, high energy density and low fat’ DP z-scores and cardiometabolic risk factors in adolescents aged 13 years recruited from three southern states of Peninsular Malaysia

Cardiometabolic risk factors*	Male, *n* = 173		Female, *n* = 412		Total, *n* = 583^a^	
	2nd tertile vs 1st tertile	3rd tertile vs 1st tertile	2nd tertile vs 1st tertile	3rd tertile vs 1st tertile	2nd tertile vs 1st tertile	3rd tertile vs 1st tertile
Overweight/Obese, *n* = 452	1.2 (0.5, 3.1)	0.6 (0.2,1.8)	1.0 (0.5,1.8)	0.9 (0.5,1.6)	1.0 (0.6,1.7)	0.9 (0.5,1.4)
Abdominal Obesity, *n* = 452	0.9 (0.2,4.3)	0.9 (0.2,4.5)	0.8 (0.3,1.8)	0.5 (0.2,1.2)	0.9 (0.4,1.9)	0.7 (0.3,1.6)
Dyslipidaemia, *n* = 283	0.9 (0.1,8.8)	3.2 (0.5119.0)	2.1 (0.9,4.6)	2.2 (0.9,5.0)	2.0 (0.9,4.2)	2.7* (1.3,5.6)
Elevated blood glucose (≥5.60 mmol/L), *n* = 283	0.0 (0.0,0.0)	0.0 (0.0,0.0)	0.0 (0.0,0.0)	0.5 (0.1,4.2)	1.0 (0.25.9)	3.2 (0.7,15.4)
Elevated total cholesterol (≥5.20 mmol/L), *n* = 283	0.9 (0.1,8.8)	3.2 (0.5119.0)	2.1 (0.9,4.6)	2.2 (0.9,5.0)	2.0 (0.9,4.2)	2.7* (1.3,5.6)
Lower HDL-cholesterol level (≤1.03 mmol/L), *n* = 283	0.0(0.0,0.0)	1.2 (0.1,19.0)	0.0 (0.0,0.0)	0.5 (0.1,3.0)	0.0 (0.0,0.0)	1.2 (0.3,4.1)
Elevated LDL-cholesterol (≥4.12 mmol/L), *n* = 283	1.5(0.4,5.2)	3.2 (0.9,11.2)	1.6 (0.8,3.3)	1.5 (0.7,3.1)	1.7 (0.9,3.0)	1.9* (1.0,3.5)
Elevated triglycerides (≥1.70 mmol/L), *n* = 283	0.0 (0.0,0.0)	0.2 (0.0,31.7)	1.6 (0.1,19.1)	3.7 (0.4,37.1)	0.6 (0.1,3.7)	2.1 (0.4,9.8)
Elevated serum insulin (≥25.0 uIU/mL), *n* = 283	1.1 (0.0,25.7)	9.8 (0.4222.8)	0.5 (0.1,2.2)	0.6 (0.1,2.5)	0.6 (0.2,2.0)	1.2 (0.4,3.6)
Abnormal HOMA-IR level (≥4.0 unit), *n* = 283	1.8 (0.3,12.0)	4.6 (0.7,28.6)	1.4 (0.6,3.4)	0.9 (0.3,2.2)	1.7 (0.8,3.6)	1.3 (0.6,3.0)

Model adjusted for school location, mother’s educational level, dietary misreporting, physical activity and BMI (for blood biomarker only) ^a^Model adjusted for sex, school location, mother’s educational level, dietary misreporting, physical activity and BMI (for blood biomarker only). *p < 0.05 is considered significant. *HDL-C* High Density Lipoprotein Cholesterol, *LDL-C* Low Density Lipoprotein Cholesterol, *HOMA-IR* Homeostatic Model Assessment of Insulin Resistance

## Discussion

The identified ‘high sugar, high fibre, high energy density and low fat’ DP in this study was associated with higher odds of having elevated TC, LDL-C levels and dyslipidaemia in adolescents aged 13 years in Malaysia.

Despite the similarities on the DP assessment (RRR) and response variables, DP identified in this study differed from those characterised in a previous study among the UK children, explicitly the Avon Longitudinal Study of Parents and Children (ALSPAC) [14]. In the ALSPAC study, two DPs namely the ‘high energy density, high sugar, high fat and low fibre’ DP and ‘non-energy density, high sugar, low fat’ DP were identified. Nevertheless, the DPs identified in the current study as well as in the ALSPAC study were characterised mainly by foods high in sugar and therefore suggesting major role of sugar in an adolescent’s diet, at least in the studies from these two countries.

The identified DP in this study was characterised by high consumption of SSB, fruit and sweet with lower consumption of cereal and cereal-based dishes, as well as, meat and meat dishes. This is in line with a review on added sugar intake among Malaysian adolescents whereby a high frequency of sugary foods and beverages intake for breakfast and during snacking times was reported [42]. The common sweetened foods and beverages frequently consumed (more than twice a week) by Malaysian adolescents include carbonated drinks, fruit juices, caffeinated and chocolate drinks, biscuits, candies, ice cream and cakes [43]. On the other hand, a higher SSB intake among Malaysian adolescents aged 13 years in Kuala Lumpur was found to be associated with increased levels of FBG, TG, insulin and HOMA-IR and decreased HDL-C level [44]. The excessive intake of sugar (sucrose, fructose, high fructose corn syrup) can directly affect the regulation of lipid and carbohydrate metabolism or it can affect the metabolism indirectly by promoting continuous positive energy balance and gradual weight gain over the long term [45].

The findings from this study were in line with a recent review that have evaluated the effect of dietary sugar on cardiometabolic risk in adults [46]. It was demonstrated that the replacement of fat intake with refined carbohydrate was associated with elevated blood lipids and blood pressure [46, 47]. This is a particular concern as refined carbohydrates (added sugar and SSB) have been also linked to the risk of heart disease due to its large consumption [48, 49]. Although it is quite natural to experience peaks in TC and LDL-C levels during adolescence, an elevated LDL-C during adolescence was a significant predictor for 38% of dyslipidaemia cases in adulthood [50].

The positive correlation observed between the identified DP and higher fruit intakes contradicted findings from the Western countries [11, 14, 51]. Nevertheless, this correlation is consistent with the pattern of fruit intakes in other studies conducted among the Malaysian adolescents [52, 53]. For instance, a cross-sectional namely the South East Asian Nutrition Surveys (SEANUTS Malaysia) study reported that a total of 1773 children aged between 7 to 9 years reported to have achieved 14% of the recommended daily fruit intake (2 servings), while children aged between 10 and 12 years consumed 20% as per the recommendation [52]. Another cross-sectional study in 454 adolescents aged 12 to 19 years in Kelantan, Malaysia also reported that older adolescents were more likely to choose healthy foods such as fruits, vegetables and dairy products compared to their younger counterparts [54]. Nevertheless, it is quite common in Malaysia that a few type of fruits are eaten as a fried item e.g. banana or jackfruit fritters, therefore an increase in the consumption of fruits in this study could be due to the misconception of fried fruits as a healthy food intake [43].

Fruits are known to be rich in fibre and antioxidant contents and are perceived as a healthy snack by all age groups including adolescents [55]. Evidence suggested that an increase fibre intake may provide protection against cardiometabolic risk factors in adults [56]. Although the DP identified in this study was characterised by the favourable aspect of high fibre intake from fruits, it was mainly characterised by tropical fruits available in Malaysia such as papaya, guava, pear, starfruit, mango, banana, lanzones, durian, jackfruit and *rambutan*, which are high in fructose (sugar) as compared to non-tropical fruits such as apples and oranges [57, 58]. A lower correlation coefficient between percentage of energy from total fat and the identified DP in this study may be due to the compensation of low dietary fat intakes with high free sugar intakes.

Although the selected response variables were previously linked to obesity, the identified DP was not associated with overweight or obesity and abdominal obesity in this study [12]. However, the lack of significant associations seen in the current could be due to the use of BMI and WC instead of fat mass to measure adiposity [11]. Reduction in the final sample size may be another reason for the lack of significant association between the identified DP and obesity in this study. Nonetheless, no significant differences were reported (data not shown) in the anthropometric outcomes and cardiometabolic biomarkers, as well as socio-economic characteristics, particularly maternal education and school location between the total participants and 336 adolescents (with both valid dietary data and biomarkers data) who were included in the multivariate analysis. The present study also reported no significant association between the identified DP and cardiometabolic risk factors specific to any gender, even though, males were reported to have a higher mean FBG level and a lower mean TC, HDL-C and LDL-C levels compared to females. This could be due to differences in the timing of puberty among the study adolescents [59, 60].

It is important to note that this study was the first to examine relationships between empirical DPs and cardiometabolic risk factors among Malaysian adolescents. Furthermore, the application of RRR, a-priori method to derive a specific DP and the ability to control for dietary misreporting were also the strengths of this study. Despite these study strengths, there are a few limitations worth mentioning. Firstly, this cross-sectional study was subjected to the possibility of finding significant associations by chance alone. Besides, the findings from this study could not be generalised due to limited coverage of the study location, whereby the study was focused only in the southern region of Peninsular Malaysia. Another limitation of this study is the biases inherent to the dietary assessment method. However, this limitation was minimised by using nutrient densities e.g. DED, fibre density and percentage of energy from fat and sugar as response variables so that errors linked to the dietary assessment method could be reduced [61]. Furthermore, the reduced sample size of the available valid dietary data for the RRR analysis could have influenced the strength of relationships between the identified DP and biochemical parameters. However, this is an unavoidable issue particularly among adolescents and that at this large scale of a study.

## Conclusion

A DP characterised by food intakes high in free sugars and energy density was associated with elevated lipid profiles, particularly cholesterol and LDL-C levels among adolescents aged 13 years in Malaysia. Further longitudinal studies are recommended to strengthen the role of a dietary pattern explained by food intakes high in sugar and energy density in the development of cardiometabolic risk factors in young people in order to design effective health promotion initiatives.

## Supplementary information

**Additional file 1: Figure S1.** Number of adolescents who provided anthropometric, biochemical, dietary and physical activity data **Table S1.** Food items included in the food groups selected as predictors in RRR analysis to derive ‘high sugar, high fibre, high energy density and low fat’ dietary pattern among Malaysian adolescents aged 13 years attending secondary schools **Table S2.** Characteristics of dietary patterns among Malaysian adolescents aged 13 years attending secondary schools derived using RRR analysis

## Data Availability

The datasets generated and/or analysed during the current study are not publicly available due the raw information gathered in this research were kept strictly confidential as stated in respondents’ consent agreement but are available from the corresponding author on reasonable request.

## Abbreviations

ALSPAC: Avon Longitudinal Study of Parents and Children
BMI: Body mass index
BMR: Basal metabolic rate
CVD: Cardiovascular diseases
DED: Dietary energy density
DP: Dietary pattern
EI: Energy intake
FBG: Fasting blood glucose
FFQ: Food frequency questionnaire
HDL-C: High-density lipoprotein cholesterol
HOMA-IR: Homeostasis model assessment
JKEUPM: Universiti Putra Malaysia’s ethics committee for research involving human subjects
LDL-C: Low-density lipoprotein cholesterol
PAL: Physical activity level
PAQ-C: Physical activity questionnaire for older children
Raine: The Western Australian Pregnancy Cohort Study
RRR: Reduced rank regression
SEANUTS: South East Asian Nutrition Surveys
SSB: Sugar-sweetened beverages
TC: Total cholesterol
TG: Triglycerides
WC: Waist circumference
WHO: World Health Organization
