# Supplementary material for: Associations of an empirical dietary pattern with cardiometabolic risk factors in Malaysian adolescents
Source: Nutr Metab (Lond). 2020 Apr 7;17:28. doi: 10.1186/s12986-020-00447-x (PMC7140313; doi:10.1186/s12986-020-00447-x)
Supplement: Supplementary file 1 — Additional file 1: Figure S1. Number of adolescents who provided anthropometric, biochemical, dietary and physical activity data Table S1. Food items included in the food groups selected as predictors in RRR analysis to derive ‘high sugar, high fibre, high energy density and low fat’ dietary pattern among Malaysian adolescents aged 13 years attending secondary schools Table S2. Characteristics of dietary patterns among Malaysian adolescents aged 13 years attending secondary schools derived using RRR analysis [file 12986_2020_447_MOESM1_ESM.docx]

# Supplementary information

**Figure S1. Number of adolescents who provided anthropometric, biochemical, dietary and physical activity data**

Estimated sample size

n = 1000

Adolescents with both valid dietary and anthropometric data

n = 582

Adolescents with both valid dietary and biochemical data

n = 336

Anthropometric data

N = 930

Valid dietary data

n = 585

Biochemical parameter

n = 507

Adolescents who agreed to participated

n = 933

Physical Activity

n = 793

Regression analysis

**Table S1. Food items included in the food groups selected as predictors in RRR analysis to derive ‘high sugar, high fibre, high energy density and low fat’ dietary pattern among Malaysian adolescents aged 13 years attending secondary schools**

| Food groups | Food items |
| --- | --- |
| 1. Cereal and cereal based dishes | White rice, porridge, coconut rice set, chicken rice set, fried rice, buttered rice, glutinous rice, fried rice with red meat, stir-fried rice with omelette, fried rice with chicken, fried rice noodle, stir-fried rice noodle, *laksa* noodle, rice noodle soup, rice noodle tom yum, white bread, sausage bread, sardine bread, sardine sandwich bread, fried wheat noodle, wheat noodle soup, curry wheat noodle, wheat noodle *bandung* style*, lor mee*, rice noodle *hokkien* style, instant dry noodle, *roti canai*, yellow dhal gravy serve with *roti canai*, *dosai, capati*, biscuits, chocolate cream biscuits, chocolate biscuits, chocolate cream bread |
| 2. Meat and poultry | Fried chicken, chicken soup, chicken tom yum, chili fried chicken, chicken *kurma*, chicken fried in soy sauce, chicken curry, chicken in coconut milk with chili, fried beef, *rendang* beef, beef soup, beef in coconut milk with chili, fried mutton, mutton curry, roasted mutton, mutton *kurma*, mutton in coconut milk with chili, fried pork, pork soup, pork in sweet sour sauce |
| 3. Seafood and shellfish | Fried catfish, roasted catfish, fried catfish in soy sauce, fried catfish in chilli, catfish in sour soup, fried cuttlefish in chilli, fried tilapia, roasted hardtail scad, fried hardtail scad, fried sardine in chilli, canned sardine, fried mackerel, roasted mackerel, mackerel in tamarind soup, mackerel in coconut milk with chili, mackerel curry, seabass in coconut milk, tuna in soy sauce, tuna coconut milk with chili, fried river catfish, river catfish in *tempoyak* soup, fried anchovy, fried anchovy in chilli, fried black pomfret, pomfret in *taucu* sauce, pomfret in sweet sour sauce, fried red snapper, salted fish, salted fried snakehead, fried prawn, fried flour coated prawn, fried shrimp in chilli, snail in coconut milk with chili |
| 4. Milk and dairy products | UHT full cream milk, UHT low fat milk, full cream milk powder, milk curd, ice cream, yogurt, cheese |
| 5. Egg and egg dishes | Sunny side up egg, omelette, boiled egg, fried egg in soy sauce, fried egg in chilli |
| 6. Nuts | Fried soybean cake |
| 7. Vegetables | Fried water spinach, water spinach in clear soup, fried water spinach with prawn paste, fiddlehead fern in coconut milk, fried spinach, mustard green soup, fried kale with salted fish, fried okra, fried long bean, fried bean sprout, cabbage in coconut milk, fried cabbage, fried broccoli, fried cauliflower, fried eggplant, fried eggplant in chilli, luffa in coconut milk, gourds clear soup, pumpkin in coconut milk, carrot in clear soup, fried carrot, mix vegetable soup, fried mix vegetables, spicy and sour soup (*rasam*) |
| 8. fruits | Red apples, papaya, watermelon, orange, pear, guava, starfruit, mango, banana, canned fruits, dried fruits, lanzones, durian, *rambutan*, jackfruit |
| 9. Local desserts | Onion fritters, anchovies fritters, curry puff, banana fritter, coconut pancake, fried banana, fried Chinese breadstick, steamed layered cake, net crepe, steamed sponge cake, *idli, vadai*, Chinese dumpling |
| 10. Sweet sweetened beverages | Cordial drinks, Horlicks, guava with sour plum drink, carbonated drinks, isotonic carbonated drinks, tea, ice lemon tea, tea with sweetened condensed milk (*teh tarik*), orange juice, coffee, packaged fruit drinks, coconut drink, watermelon with lychee drink, cereal drink, flavoured yogurt drink, chocolate flavoured UHT milk, coffee or strawberry flavoured UHT milk, 3 in 1 packet drinks, chocolate malted drink. |
| 11. Sweets | Granulated sugar, condensed milk, *seri kaya* spread, blueberry jam, dairy milk chocolate, crispy chocolate, sweet soy sauce, chili sauce |
| 12. Processed food | Fish burger, fried sausage, chicken nugget |
| 13. Fast foods and snacks | Kentucky Fried Chicken snack plate set, McDonalds Mc Chicken set, pepperoni pizza, chicken and pineapple pizza, beef, chicken and onion pizza, beef and onion pizza, chicken, mushroom and tomato pizza, corn-based snack, potato chips |

**Table S2. Characteristics of dietary patterns among Malaysian adolescents aged 13 years attending secondary schools derived using RRR analysis**

| DPs derived | % of variation explained | Correlation |
| --- | --- | --- |
| DP1 or known as ‘High sugar, high fibre, high energy density and low fat’ DP | | |
| All response variables | **35** |  |
| DED | 22 | 0.39 |
| Fibre density | 52 | 0.61 |
| % energy from total fat | 10 | -0.26 |
| % energy from sugar | 57 | 0.64 |
| DP2 or known as ‘Low fibre, high energy density and high fat’ DP | | |
| All response variables | **14** |  |
| DED | 55 | 0.77 |
| Fibre density | 59 | -0.33 |
| % energy from total fat | 26 | 0.54 |
| % energy from sugar | 58 | 0.06 |
| DP3 or known as ‘High fibre, low energy density, high sugar and high fat’ DP | | |
| All response variables | **9** |  |
| DED | 62 | -0.43 |
| Fibre density | 63 | 0.33 |
| % energy from total fat | 49 | 0.79 |
| % energy from sugar | 60 | 0.28 |
| DP4 or known as ‘High fibre, low sugar, high energy density and high fat’ DP | | |
| All response variables | **1** |  |
| DED | 62 | 0.25 |
| Fibre density | 66 | 0.64 |
| % energy from total fat | 49 | 0.12 |
| % energy from sugar | 64 | -0.72 |
